# Phosphoribulokinase mediates nitrogenase-induced carbon dioxide fixation gene repression in *Rhodobacter sphaeroides*

**DOI:** 10.1099/mic.0.000160

**Published:** 2015-11

**Authors:** Ryan M. Farmer, F. Robert Tabita

**Affiliations:** Department of Microbiology, The Ohio State University, 484 West 12th Avenue, Columbus, OH 43210-1292, USA

## Abstract

In many organisms there is a balance between carbon and nitrogen metabolism. These observations extend to the nitrogen-fixing, nonsulfur purple bacteria, which have the classic family of P(II) regulators that coordinate signals of carbon and nitrogen status to regulate nitrogen metabolism. Curiously, these organisms also possess a reverse mechanism to regulate carbon metabolism based on cellular nitrogen status. In this work, studies in *Rhodobacter sphaeroides* firmly established that the activity of the enzyme that catalyses nitrogen fixation, nitrogenase, induces a signal that leads to repression of genes encoding enzymes of the Calvin–Benson–Bassham (CBB) CO_2_ fixation pathway. Additionally, genetic and metabolomic experiments revealed that NADH-activated phosphoribulokinase is an intermediate in the signalling pathway. Thus, nitrogenase activity appears to be linked to *cbb* gene repression through phosphoribulokinase.

## Introduction

Homeostatic control elements regulate all aspects of an organism's complex network of metabolism; this control serves to coordinate and maintain balanced growth. The nitrogen regulatory cascade, containing well-studied members such as glutamine synthetase, P(II) and the NtrBC two-component regulatory system, is one of the elements that balances the metabolism of nitrogen sources based on the carbon and nitrogen statuses of the cell (Dixon & Kahn, 2004; Forchhammer, 2007; Masepohl & Kranz, 2009). One enzyme complex that is regulated by this cascade in nitrogen-fixing organisms is nitrogenase.

Nitrogenase is a complex of NifH, NifD and NifK peptides that, together, are responsible for catalysing the conversion of nitrogen gas into ammonia, which is an easily metabolized form of nitrogen. Typically, nitrogenase synthesis is repressed when ammonium is the nitrogen source for growth, while this enzyme complex is synthesized when organisms are grown with ‘poor’ nitrogen sources, specifically N_2_ gas or amino acids such as glutamate, as extensively studied in the nonsulfur purple (NSP) bacterium *Rhodobacter capsulatus* (Hillmer & Gest, 1977; Masepohl & Kranz, 2009). When glutamate is the nitrogen source, the nitrogenase complex is not necessary for growth, so (i) nitrogenase mutants that would normally be lethal under nitrogen-fixing conditions can be studied and (ii) all nitrogen gas can be removed from such cultures, resulting in nitrogenase catalysing only hydrogen production, thus not affecting cellular nitrogen levels (Hillmer & Gest, 1977; Hoffman *et al.*, 2013; Joshi & Tabita, 1996; Tao *et al.*, 2012).

Nitrogenase can also be used by the cell to maintain redox balance. Typically, when NSP bacterial cultures are grown photoheterotrophically, redox balance is maintained by the Calvin–Benson–Bassham (CBB) pathway, but several studies have shown strains that have an incomplete CBB pathway developed mutations that influenced expression of nitrogen fixation genes (Joshi & Tabita, 1996; Paschen *et al.*, 2001; Rey *et al.*, 2007; Tichi & Tabita, 2000, 2001; Wang *et al.*, 2011). The CBB cycle is required for photoautotrophic growth when CO_2_ is employed as the sole carbon source, but it is also required under photoheterotrophic growth conditions to maintain redox balance in the presence of organic carbon substrates (reviewed by Tabita, 1995; see also McEwan, 1994). Although required under both conditions, the expression level of the *cbb* genes is lower during photoheterotrophic growth (Gibson & Tabita, 1993; Gibson *et al.*, 2002).

For the NSP bacterium *Rhodobacter sphaeroides*, the *cbb* genes are arranged on two distinct operons with two known regulators for both operons: RegA and CbbR (Dangel *et al.*, 2005; Dubbs *et al.*, 2000; Dubbs & Tabita, 2003). RegA, a global response regulator protein, is part of a two-component system with its kinase RegB, which senses the redox state of the quinone pool (Dubbs & Tabita, 2004; Wu & Bauer, 2008, 2010). CbbR, a member of the LysR-type transcriptional regulators (LTTRs), is specific to the *cbb* operons (Dubbs *et al.*, 2000; Dangel & Tabita, 2009). The activity of LTTRs is often controlled by interactions with small molecules that are usually unique metabolites in the regulated pathway (Schell, 1993). As such, CbbR is activated by ribulose-1-5-bisphosphate (RuBP), the unique metabolite of the CBB pathway, which is the product of phosphoribulokinase (PRK) catalysis (Dangel *et al.* 2005; Joshi *et al.* 2012).

PRK enzymes are classified into two groups. Those that typically occur in anoxygenic photosynthetic organisms, such as NSP bacteria, are inhibited by AMP and allosterically activated by NADH (Gibson & Tabita, 1987; Novak & Tabita, 1999; Rindt & Ohmann, 1969). Conversely, oxygenic phototrophs, such as eukaryotes and cyanobacteria, contain PRK enzymes that are not known to respond to NADH levels. Instead, PRKs from eukaryotes are regulated by redox sensitive disulfide bonds and inhibitory complex formation with a small protein, CP12. While redox sensitive cysteines are present in the PRK from the cyanobacterium *Synechococcus elongatus* PCC 7942, the protein is only regulated *in vivo* by inhibitory complex formation with CP12 (Kobayashi *et al.*, 2003; Miziorko, 2000; Tamoi *et al.*, 1998, 2005).

Interestingly, in addition to the classical nitrogen regulatory cascade to maintain carbon and nitrogen homeostasis, NSP bacteria may have a system that influences carbon metabolism. It was observed through proteomic analyses of NSP organisms that the levels of enzymes involved in the CBB cycle were decreased when cells were cultured with poor nitrogen sources (Selao *et al.*, 2008; VerBerkmoes *et al.*, 2006). In addition, mutants that were constitutive for nitrogenase synthesis also displayed *cbb* gene repression while grown with ammonium (McKinlay & Harwood, 2010). In this study, the linkage between nitrogen metabolism and *cbb* gene repression was examined by employing *in vivo* genetic techniques to establish the role of PRK as a link between nitrogen and carbon metabolism in *R. sphaeroides*.

## Methods

### Growth conditions

Routine maintenance of *Escherichia coli* and *R. sphaeroides* was performed as described previously (Farmer *et al.*, 2014); in some cases Ormerod's media lacked the normal complement of molybdenum and was supplemented with 5 μM sodium tungstate.

## Bacterial strains and plasmids

The bacterial strains and plasmids used in this study are listed in Table 1. Standard molecular biology techniques, unless otherwise stated, were used for gene cloning and construction; oligonucleotides used are listed in Table S1 (available in the online Supplementary Material). *E. coli* strain JM109 was used for maintenance and construction of all plasmids and strains S17-1 and SM10 were used to conjugate the plasmids into *R. sphaeroides*.

**Table 1.** Strains and plasmids used in this study

Strain or plasmid	Description	Source or reference
*E. coli*		
JM109	Cloning strain	Yanisch-Perron *et al.* (1985)
S17-1	Conjugation strain, Sm^r^	Simon *et al.* (1983)
SM10	Conjugation strain, Km^r^	Simon *et al.* (1983)
*R. sphaeroides*		
2.4.1	Type strain	van Niel (1944)
2.4.1C1	Strain 2.4.1 with a chromosomal *cbbI–lacZ* fusion	This study
B214	Δ*cbbPII* derivative of strain 2.4.1	This study
15165	Δ*cbbPI* derivative of strain B214	This study
15165C1	Strain 15165 with a chromosomal *cbbI–lacZ* fusion	This study
NK10	Δ*nifK* derivative of strain 2.4.1	Farmer *et al.* (2014)
145	Δ*nifA* derivative of strain 2.4.1	This study
Plasmids		
pCR-Blunt II-TOPO	Cloning vector	Invitrogen
pJQ200mp18Km	Allelic exchange vector harbouring *sacB*,Km^r^	Farmer *et al.* (2014)
pJQdsac	Δ*sacB* vector derived from plasmid pJQ200mp18Km	This study
pVKCI	*cbbI–lacZ*-containing vector	Dubbs & Tabita (1998)
pVKCII	*cbbII–lacZ*-containing vector	Dubbs & Tabita (2003)
pBBRsm2MCS5	Broad-host-range vector, Sm^r^	Schneider *et al.* (2012)
pBBR-nifAwt	pBBRsm2MCS5 containing a *nifA* WT cassette	This study
pET-S7PRK	His-tag expression vector containing *Synpcc7942_0977*	Kobayashi *et al.* (2003)
pBBR-F2B	pBBRsm2MCS5 containing a *cbbII* promoter fusion to *cbbPII*	This study
pBBR-F2C	pBBRsm2MCS5 containing a *cbbII* promoter fusion to *Synpcc7942_0977*	This study

### Strain 15165

To construct a nonpolar PRK deletion strain from wild-type strain 2.4.1, the *cbbP_II_* gene (RSP_3267) was deleted from the genome of strain 2.4.1 to create strain B214, and then the *cbbP_I_* gene (RSP_1284) was deleted from strain B214 to create the PRK null strain 15165. To accomplish this construction, the *cbbFPT* genetic region from the *cbb_II_* operon from strain 2.4.1 was cloned into plasmid pCR-Blunt II-TOPO. Inverse amplification and infusion reactions (Clontech) were performed to construct a nonpolar deletion of the *cbbP_II_* gene by removing the entire sequence from the stop codon of *cbbF* to the stop codon of *cbbP*. This region was then cloned into the suicide vector pJQ200mp18Km, transformed into strain S17-1 and conjugated into strain 2.4.1. Exconjugates were selected for kanamycin resistance, subcultured until kanamycin-sensitive colonies appeared and then sequenced to confirm the deletion of the genes. Similar methods were employed to construct the *cbbP_I_* nonpolar deletion construct. This was then conjugated into strain B214 and screened as above to isolate the double PRK deletion strain 15165.

### Strain 145

To construct a strain harbouring a nonpolar deletion of *nifA* (RSP_0547) derived from strain 2.4.1, a *nifA* deletion fragment was constructed by ligating approximately 400 bases upstream of a putative ribosome-binding site of *nifA* to approximately 400 bases of the 3′ coding region into plasmid pCR-Blunt II-TOPO. This construct was then cloned into the suicide vector pJQ200mp18Km, transformed into strain S17-1 and conjugated into strain 2.4.1. Gene deletion strains were screened as described above.

### Strains 2.4.1C1 and 15165C1

Strains harbouring a chromosomal *cbb_I_-lacZ* promoter fusion were constructed by excising the *cbb_I_-lacZ* promoter fusion from plasmid pVKCI with *Eco*RI and ligating it into plasmid pJQdsac. This vector was then transformed into strain S17-1 and conjugated into strains 2.4.1 and 15165. Kanamycin-resistant colonies were screened for plasmid integration by sequencing the *cbb_I_* promoter regions. The plasmid pJQdsac was constructed after digestion of plasmid pJQ200mp18Km with *Eco*RI and *Kpn*I followed by ligating the large fragments together resulting in the excision of the *sacB* gene.

### Plasmids

The promoter region, cloned from approximately 400 bases upstream of the *nifA* ATG start codon from strain 2.4.1 genomic DNA, was ligated to the start codon of *nifA* via an engineered *Nco*I site and cloned into plasmid pBBRsm2MCS5 to construct a complementation vector, designated pBBR-nifAwt. For PRK complementation vectors, the entire *cbb_II_* promoter region up to the start codon from plasmid pVKCII was cloned upstream of the start codon of genes that encode PRK and inserted into plasmid pBBRsm2MCS5. The *cbbP_II_* gene encoding form-II PRK from *R. sphaeroides* was amplified from the genome of strain 2.4.1 and used to construct plasmid pBBR-F2B. The gene encoding PRK from *S. elongatus* PCC 7942 (His-tag free *Synpcc7942_0977*) was obtained from a vector derived from plasmid pET-S7PRK and used to construct plasmid pBBR-F2C.

## Enzyme assays and biochemical procedures

Acetylene reduction assays and Western immunoblot determinations were performed as previously described (Farmer *et al.*, 2014).

### β-Galactosidase activity

Cultures were harvested and cell pellets lysed as described (Farmer *et al.*, 2014). Cell extract was added to Z-buffer (50 mM sodium phosphate, pH 7; 10 mM KCl; 1 mM MgSO_4_; 5 mM β-mercaptoethanol) containing 0.8 mg ONPG ml^− 1^ in a total volume of 1 ml. A molar extinction coefficient of 3.1 × 10^3^ cm^− 1^ M^− 1^ was used to calculate the rate of production of *o*-nitrophenol from the continuously measured absorbance at 405 nm and was normalized against the amount of protein as determined using the Bradford assay (Bio-Rad). Unless otherwise stated, triplicate cultures were assayed in duplicate and the values are reported as the mean of the cultures ± sd.

### NADH quantification

Cultures were harvested as described above. NADH was determined using the EnzyChrom NAD^+^/NADH Assay kit (BioAssay Systems) as described by the manufacturer's directions, except cell pellets were sonicated in 200 μl extraction buffer. NADH levels were normalized to the amount of protein contained in the assay as described above. Unless otherwise stated, triplicate cultures were assayed in duplicate and the values are reported as averages of the cultures ± sd.

## Results

### Influence of nitrogen metabolism on *cbb* gene regulation

**Fig. 1.**
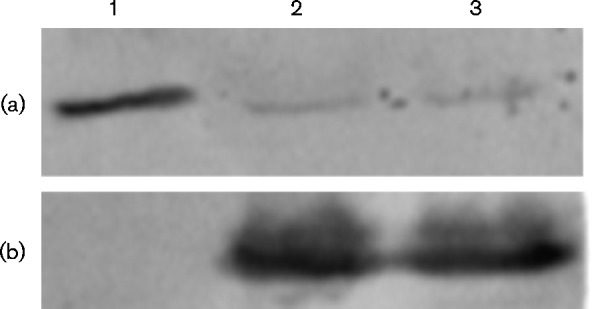
Western immunoblots of crude extracts from cultures of WT *R. sphaeroides* strain 2.4.1 grown with different nitrogen sources. Cell extracts from strain 2.4.1 grown with ammonium (lane 1), glutamate (lane 2) or nitrogen gas (lane 3) as the nitrogen source were blotted with antibodies directed against PRK (a) to visualize the co-migrating form-I and form-II PRK CBB proteins or antibodies to NifH (b) to visualize nitrogenase. The headspace of the cultures grown with ammonium or glutamate was exchanged for argon.

To determine if nitrogenase catalysis specifically, rather than nitrogen fixation in general, was sufficient to induce *cbb* gene repression, lysates were analysed from cultures of *R. sphaeroides* strain 2.4.1 grown with ammonium or nitrogen gas as the sole nitrogen source and compared to cultures grown with glutamate as the nitrogen source. For the glutamate cultures, N_2_ in the head space was exchanged for argon. Because of this exchange, the hydrogenase activity of nitrogenase catalyses proton reduction resulting in hydrogen evolution (Hillmer & Gest, 1977); therefore, the reduction of N_2_ to ammonia does not occur since the substrate for nitrogen fixation (N_2_) has been removed. With glutamate as nitrogen source, nitrogenase was synthesized at high levels (Fig. 1). Clearly, levels of the CBB cycle protein PRK were highest in lysates from cultures of WT strain 2.4.1 grown with ammonium as the nitrogen source compared to cultures grown with nitrogen gas or glutamate (Fig. 1). It is also apparent that *cbb* gene repression occurred regardless of whether nitrogenase catalysed nitrogen fixation or just hydrogen production.

**Fig. 2.**
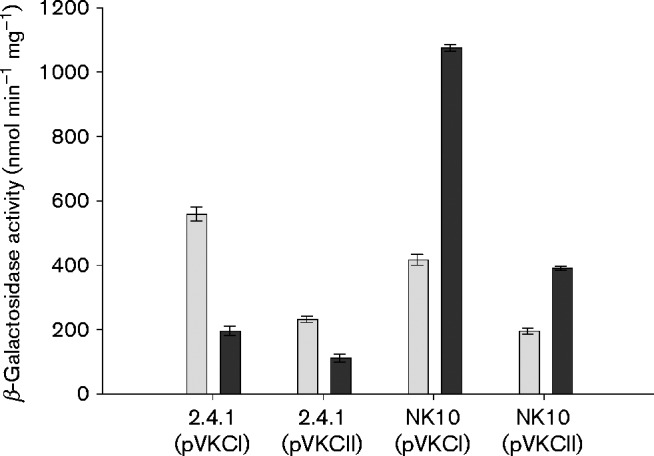
Plasmid-based *cbb* promoter fusion activities from lysates of *R. sphaeroides* WT strains 2.4.1 and nitrogenase null mutant strain NK10. β-Galactosidase activities were measured from lysates of strains 2.4.1 or NK10 complemented with plasmids pVKCI or pVKCII containing the *cbbI*– or *cbbII*–*lacZ* promoter fusions, respectively. Nitrogen sources for the cultures were either ammonium (light bars) or glutamate (dark bars).

Western immunoblots could not distinguish between form-I PRK synthesized and encoded by the *cbbP_I_* gene of the *cbb_I_* operon or form-II PRK synthesized and encoded by *cbbP_II_* of the *cbb_II_* operon. Thus, *lacZ* fusions to either the *cbb_I_* or *cbb_II_* promoter were employed and β-galactosidase levels determined from extracts of strains containing each reporter gene fusion as a measure of *cbbP_I_* or *cbbP_II_* gene expression (Fig. 2). For glutamate-grown WT strain 2.4.1, *cbb_I_* and *cbb_II_* promoter activities both decreased relative to extracts from ammonium-grown cells. Additionally, when both *cbb* promoter fusion activities were measured in nitrogenase null strain NK10 grown with glutamate, β-galactosidase levels were significantly increased over those of ammonium-grown cultures, especially *cbbP_I_* promoter activity (Fig. 2). Also, the *cbb_I_* promoter activities from ammonium grown strains 2.4.1 and NK10 were similar, as were the *cbb_II_* promoter activities, signifying that aberrant regulation did not occur in the nitrogenase-null strain when ammonium was used as the nitrogen source.

**Fig. 3.**
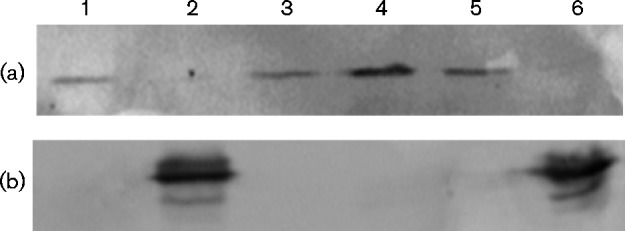
Western immunoblot of crude extracts from cultures of *R. sphaeroides nifA* strain 145. Cell extracts from ammonium-grown cultures (lanes 1, 3 and 5) or glutamate-grown cultures (lanes 2, 4 and 6) were blotted with antibodies directed against PRK (a) to visualize the co-migrating CBB proteins form-I and form-II PRK or NifH (b) to visualize nitrogenase. Samples were collected from WT strain 2.4.1 (lanes 1 and 2), *nifA* strain 145 (lanes 3 and 4) and *nifA* strain 145 (pBBR-nifAwt) (lanes 5 and 6).

Comparisons of strains 2.4.1 and an additional nitrogenase null strain, strain 145, which contains a nonpolar deletion of the *nif* operon transcriptional activator gene, *nifA*, also supported these observations. Western immunoblots of PRK from lysates of strain 145 showed an increase in *cbb* expression when this strain was grown with glutamate (Fig. 3, lanes 3 and 4) and also showed that upon complementation with the WT *nifA* gene, both PRK and NifH protein levels returned to that of strain 2.4.1 (Fig. 3, lanes 5 and 6).

**Fig. 4.**
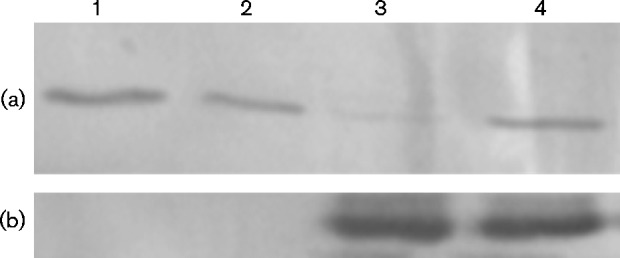
Western immunoblot of crude extracts from cultures of strain 2.4.1 grown with tungsten. Cell extracts of strain 2.4.1 grown with ammonium (lanes 1 and 2) or glutamate (lanes 3 and 4) were blotted with antibodies directed against PRK (a) to visualize the co-migrating CBB proteins form-I and form-II PRK or NifH (b) to visualize nitrogenase. Lanes 2 and 4 contained media without the normal complement of molybdenum which was supplemented with 5 μM sodium tungstate.

Finally, when the nitrogenase inhibitor tungsten replaced molybdenum in the growth media of wild-type strain 2.4.1 cultures, PRK levels no longer were repressed in cells grown with glutamate (Fig. 4). To verify nitrogenase inhibition, residual nitrogenase activity was less than 10 % of that from molybdenum grown cultures, as detected by the acetylene reduction method (data not shown). These results all indicated that nitrogenase activity leads to repressed *cbb* gene expression.

### Involvement of PRK in CBB regulation

Strain 15165 is a CBB null strain derived from strain 2.4.1 that contains nonpolar deletions of the *cbbP* genes from both *cbb* operons. For unknown reasons, this strain exhibited a high rate of recombination as observed after sequencing plasmids that have been conjugated into this strain; however, isolates were obtained that could stably maintain a *cbb_I_-lacZ* fusion integrated into the chromosome; e.g. strain 15165C1. Strains 15165 and 15165C1 were also able to stably maintain pBBR-F2B or pBBR-F2C as plasmids containing the genes encoding form-II PRK from *R. sphaeroides* or PRK from *S. elongatus* PCC 7942, respectively. Strains 15165 and 15165 (containing the empty plasmid pBBRsm2MCS5) required the addition of an alternative electron acceptor, DMSO, in order to grow photoheterotrophically with ammonium as nitrogen source. By contrast, strain 15165 complemented with plasmids pBBR-F2B or pBBR-F2C did not require DMSO addition. Additionally, strains 15165 (pBBR-F2B) and 15165 (pBBR-F2C) grew with similar doubling times (Table 2), indicating that the PRK isozyme from the cyanobacterium *S. elongatus* PCC 7942 complemented strain 15165 as well as the *R. sphaeroides* PRK.

**Table 2.** Generation times (in hours) of PRK complementation strains

Strain	Nitrogen source	
	Ammonium	Glutamate
15165	NG*	9.7 ± 0.5
15165 (pBBRsm2MCS5)	NG	11.0 ± 0.6†
15165 (pBBR-F2B)	7.1 ± 0.1	12 ± 1
15165 (pBBR-F2C)	7.5 ± 1.3	12 ± 2

* NG, no growth without the addition of DMSO.

† Average ± range for two samples, all other values were derived from at least triplicate samples.

**Fig. 5.**
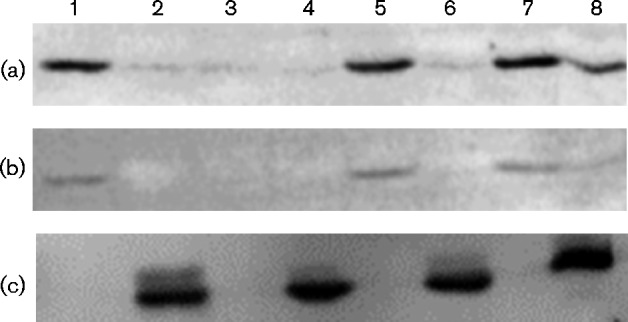
Western immunoblot of crude extracts from cultures of *cbbPI/cbbPII* null strain 15165. Cell extracts were blotted with antibodies directed against form-I RubisCO (a), form-II RubisCO (b) or NifH (c). Lanes 1 and 2 contained extracts from WT strain 2.4.1 grown with ammonium or glutamate respectively; all other lanes contained extracts from strain 15165. Lanes 3 and 4 contained extracts from strain 15165 grown with ammonium/DMSO or glutamate respectively. Lanes 5 and 6 contained extracts from strain 15165(pBBR-F2B) complemented with the *R. sphaeroides cbbPII* gene and grown with ammonium and glutamate, respectively. Lanes 7 and 8 contained extracts from strain 15165 (pBBR-F2C) complemented with the cyanobacterial (*S. elongatus*) *prk* gene and grown with ammonium or glutamate, respectively.

**Fig. 6.**
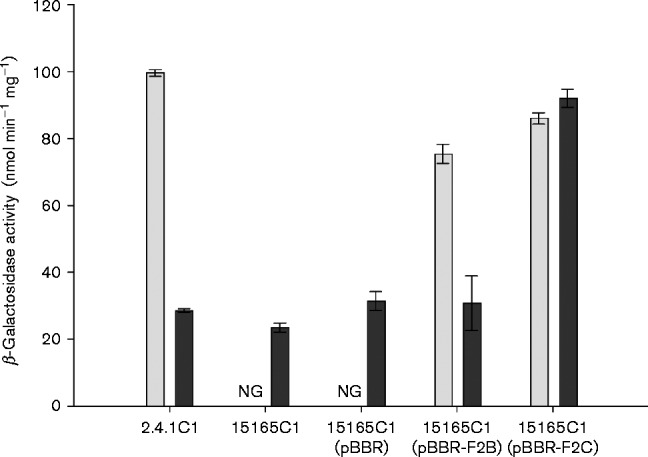
Chromosomal-based *cbb_I_* promoter fusion activities from lysates of strains 2.4.1C1 and 15165C1. β-Galactosidase activities were measured from lysates of strains 2.4.1C1 and 15165C1 grown with ammonium (light bars) or glutamate (dark bars). No growth (NG) occurred for the PRK-null strain (15165C1) with or without the empty vector (pBBR) when cultured with ammonium.

Western immunoblots were used to detect the presence of the additional CBB proteins form-I and form-II RuBP carboxylase/oxygenase (RubisCO) because strain 15165 does not contain the genes that encode PRK. Form-I RubisCO is encoded by the *cbbLS* genes (RSP_1282 and RSP_1281 respectively) of the *cbb_I_* operon and form-II RubisCO is encoded by the *cbbM* gene (RSP_3271) of the *cbb_II_* operon; therefore, the protein abundances of each isozyme can be used to distinguish regulation of each operon. Both Western immunoblots (Fig. 5) and *cbb_I_-lacZ* promoter activities (Fig. 6) showed that extracts from strains 15165 or 15165C1, when complemented with *cbbP_II_*, had *cbb* expression levels similar to those of WT strain 2.4.1. Interestingly, upon complementation with plasmid pBBR-F2C, encoding the cyanobacterial PRK, strains 15165 and 15165C1 exhibited *cbb* expression levels that were greater than strain 2.4.1 when grown with glutamate, but *cbb* expression was similar when grown with ammonium (Figs. 5 and 6). Although the Western blot would indicate differential expression between *cbb_I_* and *cbb_II_* operons compared with cells containing the cyanobacterial PRK, a stable *cbb_II_* promoter *lacZ* chromosomal fusion could not be made to further validate the Western blot results. These results indicated that the cyanobacterial PRK isozyme abrogated the nitrogenase-induced repression of *cbb* expression for the *cbb_I_* operon and partially for the *cbb_II_* operon.

### NADH levels

To determine if the intracellular levels of NADH changed upon nitrogenase expression, NADH pools were measured with the BioAssay Systems EnzyChrom kit. Both WT strain 2.4.1 and *nifH* null strain NK10 had 0.3 nmol of NADH per mg of protein (with sd of 0.1 and 0.05, respectively) when grown with ammonium. When grown with glutamate, strain NK10 contained 2.1 ± 0.1 nmol of NADH per mg protein, which is three times higher than the levels of strain 2.4.1, which had 0.7 ± 0.2 nmol of NADH per mg protein. For unknown reasons, it was noticed that spiked samples measured inconsistently when results were compared from samples of cultures grown with different nitrogen sources; therefore reliable comparisons could only be made among samples from cultures grown with the same nitrogen source.

## Discussion

Although many metabolic pathways have been determined, in many instances, how these pathways are regulated and integrated with total cellular metabolism remains to be elucidated. This is because regulation is complex and often arises in unexpected circumstances. Studies of NSP bacterial cultures grown photoheterotrophically with various nitrogen sources led to observations that the nitrogen source affects carbon metabolism, specifically the CBB cycle (Edgren & Nordlund, 2004; Selao *et al.*, 2008; VerBerkmoes *et al.*, 2006). Many studies have shown the effects upon nitrogen metabolism due to carbon status, but very few have described the inverse of how carbon metabolism responds to nitrogen status (Doucette *et al.*, 2011; Ninfa, 2007).

In *R. sphaeroides*, it is clear that nitrogenase activity leads to repression of *cbb* gene expression and the results of the current study show that repression occurred regardless of whether nitrogenase was actively fixing nitrogen or whether it just catalysed the reduction of protons, since repression was observed even when no nitrogen gas was present in the culture. While previous studies in a variety of NSP bacteria have indicated that conditions that favoured nitrogenase synthesis favoured *cbb* gene repression (McKinlay & Harwood, 2010; Smith & Tabita, 2002; Tichi & Tabita, 2001), the current study specifically implicated nitrogenase activity in *cbb* repression.

PRK activity was hypothesized to be required for expression of the *cbb* genes because its catalytic product, RuBP, activates CbbR, the master regulator required for *cbb* transcription (Dangel *et al.*, 2005; Gibson & Tabita, 1993; Joshi *et al.*, 2012; Smith & Tabita, 2002; Tichi & Tabita, 2001). This is supported by the observations that PRK-null strains cannot grow photoheterotrophically with malate as the carbon source. Under this growth mode, the CBB cycle is required to maintain redox balance or alternative electron sinks need to be supplied (Laguna *et al.*, 2011). When DMSO was supplied as an electron sink or when the growth mode was changed to derepress the nitrogenase-catalysed reduction of protons, the PRK-null strain could grow. Additionally, Western blots showed very low levels of CBB proteins when the PRK-null strain was grown with ammonium and DMSO. It is also interesting to note that WT and PRK-null strains had similar *cbb* expression levels when grown with glutamate, which may represent a low-level background or basal expression of the *cbb* operons.

Complementation experiments of PRK deletion strains, 15165 and 15165C1, were conducted to determine if PRK activity linked nitrogenase activity with the observed *cbb* gene repression. Even though bacterial and cyanobacterial PRK have low sequence conservation and are arranged in different oligomeric states, they still catalyse the same reaction (Tabita, 1988). Therefore, it was not surprising that a comparison of the growth rates indicated that each isozyme complemented growth equally well. Additionally, analysing the phenotypes of these complemented strains allowed conclusions to be made about the impact of PRK activity and regulation on *cbb* expression because cyanobacterial PRK activity is regulated differently than PRK from *R. sphaeroides* (Kobayashi *et al.*, 2003; Miziorko, 2000; Novak & Tabita, 1999; Tamoi *et al.*, 1998, 2005; Tabita, 1988). Complementation with cyanobacterial PRK abolished nitrogenase-induced repression of the *cbb_I_* operon in PRK deletion strains but did not completely revert *cbb_II_* operon repression. These results indicated that PRK serves as a link between nitrogenase activity and *cbb* gene expression and also that additional repressive effects may influence the *cbb_II_* operon. Differential regulation of the two *cbb* operons has been previously observed in different contexts in *R. sphaeroides* (Gibson & Tabita, 1993; Gibson *et al.*, 2002; Jouanneau & Tabita, 1986), although the molecular mechanism that is responsible for this difference is currently unknown, especially in regard to photoheterotrophic growth.

Further determination of the molecular mechanism between nitrogenase activity and *cbb* gene repression requires establishing a link between nitrogenase activity and PRK regulation. As initially proposed by McKinlay & Harwood (2010), nitrogenase catalysis may indirectly result in the decrease of the NADH pool size via the *Rhodobacter* nitrogen fixation (RNF) complex, which may then affect *cbb* gene expression. The RNF complex coordinates the oxidation of NADH and the reduction of ferredoxin using reverse electron flow coupled to the proton motive force (Biegel *et al.*, 2011). Ferredoxin then donates the electrons to the NifH protein of nitrogenase (Ludden, 1991). Therefore, nitrogenase catalysis could decrease the pool size of NADH. Since NADH is known to be required for activation of PRK from *R. sphaeroides* (Gibson & Tabita, 1987; Novak & Tabita, 1999; Rindt & Ohmann, 1969), then PRK may not be as active during nitrogenase catalysis.

The hypothesis that NADH cellular pool sizes might coordinate nitrogenase activity with *cbb* repression was supported by the complementation studies using different PRK isozymes, because the activity of cyanobacterial PRK is not known to be regulated by NADH. In further support for the NADH hypothesis, the nitrogenase null strain NK10 had higher levels of *cbb* expression and also possessed higher levels of NADH upon growth with glutamate. Presumably, the increased levels of NADH might then stimulate PRK activity to catalyse the production of more RuBP, which is a positive effector for CbbR, thus causing the observed increase in *cbb* gene expression. Whether this is true will depend on future experiments as the method of ‘fixing’ the metabolite levels in the cell might need to be optimized and other methods that allow for determining NADH levels when growth occurs with different nitrogen sources will need to be developed. For example, previous studies have attempted to measure changes in NADH levels upon nitrogenase induction, but these studies were inconsistent with each other (Haaker *et al.*, 1974; Nordlund & Hoglund, 1986; Norén & Nordlund, 1994). Additionally, it will be important to develop sensitive methods to correlate these results with intracellular pool sizes of other metabolites, such as RuBP.

In conclusion, through gene deletion and tungsten inhibition studies, the activity of the nitrogenase complex was determined to be responsible for initiating repression of the *cbb* genes. Also, through gene deletion and complementation studies, *R. sphaeroides* PRK appeared to be part of a regulatory link between nitrogenase activity and *cbb* gene expression. These studies also provide additional support for differential control of the *cbb* operons in *R. sphaeroides* (Dubbs & Tabita 2003; Gibson *et al.* 2002). Finally, additional studies should be undertaken to determine what other factor(s) causes the repressive effect on the *cbb_II_* operon and whether NADH is also part of this regulatory chain.

## Supplementary Data

87 Supplementary Data
