# Supplementary material for: Phosphoribulokinase mediates nitrogenase-induced carbon dioxide fixation gene repression in Rhodobacter sphaeroides
Source: Microbiology (Reading). 2015 Nov;161(Pt 11):2184–91. doi: 10.1099/mic.0.000160 (PMC4806589; doi:10.1099/mic.0.000160)
Supplement: Supplementary file 1 — Supplementary Data [file 000160.pdf]

Table S1. Oligonucleotides used in this study.

Name	5'-3' sequence	Construct
F PRKa in spe	CGCCACTAGTCGAAGCCGATCC	*cbbFPA* genetic region from cbb_I
R PRKa in xba	GAGGTCTAGACGTGCCGGATCG	
F PRKa ds fus	GGAGCTGATCCGGACCCGGGGC	Inverse primers for $cbbP_I$ deletion
R PRKa us fus	GTCCGGATCAGCTCCGGAACAGGCC	
F PRKb in spe	TCGTACTAGTTTGCGATCAACGCCTC	*cbbFPT* genetic region from cbb_{II}
R PRKb in xba	CCGGTCTAGACCTCGATCCCCTC	
F PRKb ds fus	GGAGTTAAGGCGCGACAGACAGACGGAG	Inverse primers for $cbbP_{II}$ deletion
R PRKb us fus	TCGCGCCTTAACTCCGGAACAGGCCCCG	
F Rs 548 T speI	GTACTAGTGCAAGGTCCTGCAGGAG	Upstream HRS* for *nifA* deletion†
R nifA pro ncoI	GTCCATGGCCAGACCTCCGT	
F nifA T sacI	GCGAGCTCGAGAACTGCATC	Downstream HRS for *nifA* deletion
R nifA T xbaI	GGCTCTAGATTTGTCGCACCCC	
F FII pro xba fus	AATTGGGCCCTCTAGACAACGGTCCGCCGACAAG	cbb_{II} promoter
R FII pro nco fus	TCTTCGCCATGGCTCCTCCTGCCTCTG	
F PRKb nco fus	GAGCCATGGCGAAGAAATATCCCATCATTTCCGTGG	$cbbP_{II}$ coding region
R PRKb hind fus	GCTATGCATCAAGCTTGCCTCAGGCCC	
R PRK 6301 nco	GAAGGTCGTCCCATGGGCAAG	*Synpcc7942_0977* coding region
F PRK 6301 hind	GGATGAAAGCTTGAGCAACCTAGACGC	

*HRS, homologous recombination site

†an incomplete amplification product was ultimately used in which the 3' primer sequence plus an additional 3 base pairs were missing
